# Stem Cells from the Apical Papilla: A Promising Source for Stem Cell-Based Therapy

**DOI:** 10.1155/2019/6104738

**Published:** 2019-01-29

**Authors:** Jun Kang, Wenguo Fan, Qianyi Deng, Hongwen He, Fang Huang

**Affiliations:** ^1^Institute of Stomatological Research, Sun Yat-sen University, Guangdong Provincial Key Laboratory of Stomatology, Guangzhou 510080, China; ^2^Department of Pediatric Dentistry, Guanghua School of Stomatology, Hospital of Stomatology, Sun Yat-sen University, Guangzhou 510055, China

## Abstract

Stem cells are biological cells that can self-renew and can differentiate into multiple cell lineages. Stem cell-based therapy is emerging as a promising alternative therapeutic option for various disorders. Mesenchymal stem cells (MSCs) are multipotent adult stem cells that are isolated from various tissues and can be used as an alternative to embryonic stem cells. Stem cells from the apical papilla (SCAPs) are a novel population of MSCs residing in the apical papilla of immature permanent teeth. SCAPs present the characteristics of expression of MSCs markers, self-renewal, proliferation, migration, differentiation, and immunosuppression, which support the application of SCAPs in stem cell-based therapy, including the immunotherapy and the regeneration of dental tissues, bone, neural, and vascular tissues. In view of these properties and therapeutic potential, SCAPs can be considered as promising candidates for stem cell-based therapy. Thus the aim of our review was to summarize the current knowledge of SCAPs considering isolation, characterization, and multilineage differentiation. The prospects for their use in stem cell-based therapy were also discussed.

## 1. Introduction

**Figure 1:**
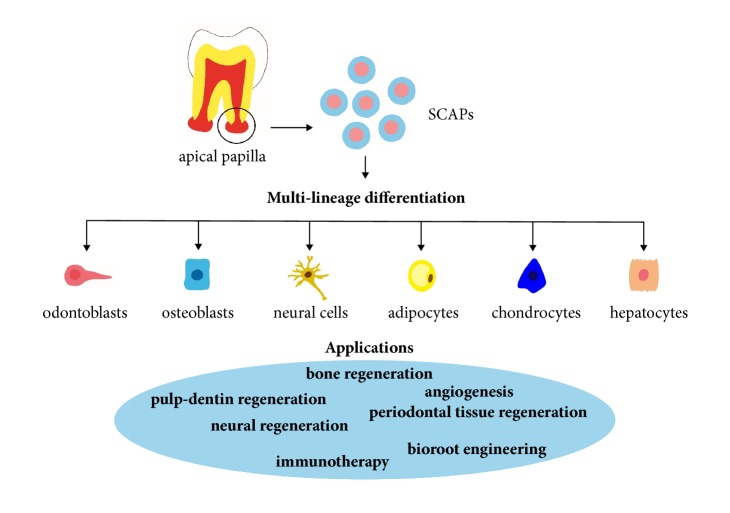
Sources, multilineage differentiation capacity, and potential applications of SCAPs.

Stem cells are biological cells that can self-renew and can differentiate into multiple cell lineages. Mesenchymal stem cells (MSCs) are multipotent adult stem cells that are isolated from various tissues. Recently, dental-tissue-derived MSC-like populations have been isolated and characterized. Stem cells from the apical papilla (SCAPs) residing in the apical papilla of immature permanent teeth represent a novel population of dental MSCs that possesses the properties of high proliferative potential, the self-renewal ability, and low immunogenicity [1]. Moreover, considerable evidence indicates that SCAPs are capable of giving rise to various lineages of cells, such as osteogenic, odontogenic, neurogenic, adipogenic, chondrogenic, and hepatogenic cells, which can be as a promising source for stem cell-based therapy (Figure 1) [1–4]. With the discovery of stem cells and the development of stem cell technology, stem cell-based therapy is emerging and moving rapidly into clinical application, which aims to replace or repair damaged cells and tissue in numerous diseases.

The aim of our review was to summarize the basics of biology of SCAPs, and the prospects for their use in stem cell-based therapy were also discussed.

## 2. Isolation of SCAPs

Recently, a variety of dental MSCs have been isolated, including dental pulp stem cells (DPSCs), stem cells from the human exfoliated deciduous teeth, SCAPs, dental follicle stem cells (DFSCs), and periodontal ligament stem cells (PDLSCs). In 2006, SCAPs were first discovered and isolated from the apical papilla tissue of incompletely developed tooth by Sonoyama et al. [1]. The apical papilla refers to the soft tissue that is loosely attached to the apices of immature permanent teeth and can be easily detached with a pair of tweezers [2]. There is a cell rich zone lying between the apical papilla and the pulp, and the apical papilla is different from the pulp in terms of containing less cellular and vascular components than the pulp [2]. However, a previous study has provided evidence that the apical papilla contains a higher number of MSCs than mature dental pulp tissue [1]. Currently, there are two common approaches to isolate and culture SCAPs. The first method is enzyme digestion. The apical papilla tissue is separated from the tip of the root, minced into pieces, and then digested in a solution of collagenase type I and dispase with gentle agitation. After digestion, tissue clumps are collected and passed through a cell strainer to obtain single cell suspension of SCAPs, which is then seeded in culture dishes [2]. Another method is explant culture, in which the apical papilla tissue is cut into samples about 1 mm^3^ in size and then plated on culture dishes [5]. Both methods can effectively isolate and culture SCAPs, but the former is more commonly used. Meanwhile, a noteworthy fact is that SCAPs can only be isolated at a certain stage of tooth development, because apical papilla evolves into dental pulp during the formation of crown and root. Since Ding et al. have confirmed that cryopreservation does not affect the biological and immunological properties of SCAPs [6]; SCAPs can be stored by cryopreservation to retain their regenerative potential for future clinical applications.

## 3. Characterizations of SCAPs

There is a large volume of published studies describing that SCAPs, like other MSCs, express the MSC-associated markers and are capable of self-renewal, proliferation, and multilineage differentiation [1]. Comparative analyses indicate that SCAPs exhibit a higher proliferation rate than DPSCs and PDLSCs [1, 2, 7, 8] but display a lower proliferation rate than DFSCs [3]. When stimulated with human platelet lysate, epiregulin, tumor necrosis factor *α*, or basic fibroblast growth factor (bFGF), SCAPs show a significantly increased proliferation rate [9–11]. In addition, compared with DPSCs, SCAPs have greater migration ability assessed by scratch assay [1]. Several studies have investigated that a variety of chemotactic factors, including stromal cell-derived factor 1, transforming growth factor *β* 1, platelet-derived growth factor, granulocyte colony-stimulating factor, and FGF 2, could promote the migration of SCAPs. Therefore, these factors may be used clinically in cell homing-based regenerative endodontic procedures in the future [12–15].

SCAPs are also characterized by the expression of surface and intracellular molecules (Table 1). Similar to other MSCs, SCAPs express STRO-1 and CD146 that are recognized as early MSCs markers [1]. They also express pluripotent markers such as octamer binding transcription factor-3/4, sex determining region Y-box 2, and nanog homeobox [3, 16]. In addition, several authors have reported the expression of a range of markers on SCAPs, including CD13, CD24, CD29, CD44, CD49, CD51, CD56, CD61, CD73, CD90, CD105, CD106, CD166, NOTCH3, and vimentin [1, 3, 16–20]. Meanwhile, SCAPs are found to be negative for the expression of CD14, CD18, CD34, CD45, CD117, and CD150, indicating that they are not of hematopoietic origin [1, 20]. Among these molecular markers, CD24 may be used to distinguish SCAPs from DPSCs and predict the differentiation of SCAPs, since it is undetectable in DPSCs [1]. As for other markers, it seems to be expressed in both SCAPs and other MSCs, so specific markers need to be further developed. Moreover, SCAPs have a higher expression of antiapoptotic protein survivin, longer telomere length, and greater telomerase activity associated with cellular lifespan and cell proliferation than DPSCs do [1, 21].

Aside from these surface and intracellular molecules, the secretome of SCAPs has also been extensively profiled. The evidence indicates that a total of 2,046 proteins are released, including chemokines, angiogenic, immunomodulatory, antiapoptotic, neuroprotective factors, and extracellular matrix proteins. Significantly, SCAPs secrete more chemokines, neurotrophins and proteins involving in metabolic processes and transcription compared to bone marrow mesenchymal stem cells (BMMSCs) [22].

SCAPs are a heterogeneous population of cells, which contain subpopulations of cells with different phenotypes and characteristics [2]. For example, the STRO-1 (pos)/CD146 (pos) subpopulation shows a higher proliferation rate and an enhanced odontogenic differentiation potential than other subpopulations [16]. However, the causes of cellular heterogeneity are still unknown, so further studies are required.

## 4. Multilineage Differentiation

Over the past 10 years, numerous studies have confirmed that SCAPs possess the capacity to differentiate into multiple cell types such as osteoblasts, odontoblasts, neural cells, adipocytes, chondrocytes, and hepatocytes.

### 4.1. Osteo/Odontogenic Differentiation

Many studies have demonstrated that SCAPs are capable of differentiating into osteoblasts and odontoblasts [1, 2, 19, 20, 23]. After culture in osteo/odontogenic medium containing L-ascorbate-2-phosphate, dexamethasone, and *β*-glycerophosphate, SCAPs are found to express specific markers of osteoblasts or odontoblasts, such as alkaline phosphatase, runt-related transcription factor 2, osteocalcin, dentin sialophosphoprotein, bone sialoprotein, and dentin matrix protein 1 [3, 7, 16, 19, 20, 23–36]. They also form mineralized nodules which can be identified by alizarin red staining for calcium deposits [1–3, 23]. Furthermore, there are a large number of studies investigating the influence of molecules on the directed differentiation of SCAPs. The osteo/odontogenic differentiation of SCAPs can be promoted by forkhead c2 [37], bone morphogenetic protein 2 ( BMP2) [37–39], BMP9 [32, 40], SH3 and multiple ankyrin repeat domains 2 [25], GATA binding protein 4 [41], 17 *β*-estradiol [28], nuclear factor I-C [42, 43], secreted frizzled-related protein 2 (SFRP2) [44, 45], WD repeat domain 63 [34], insulin-like growth factor-1 [30, 46], recombinant human plasminogen activator inhibitor-1 [26], Rac1 gene [31], early growth response gene 1 [47], sirtuin 1 [48], potassium phosphate monobasic [49], canonical NF-kappaB signaling pathway [27], wnt/*β* -catenin signaling [50], and some dentin-derived proteins [51]. By contrast, microRNA hsa-let-7b [52] and sonic hedgehog signaling [53] are able to inhibit this differentiation of SCAPs. In addition, homeobox (HOX) genes play important roles in the differentiation regulation of SCAPs. The results of investigations indicate that HOXB7 [35], distal-less homeobox 2 [54], and MEIS2 [55] promote osteogenic differentiation of SCAPs, whereas HOXC10 [36] inhibits this differentiation* in vitro*.

### 4.2. Neurogenic Differentiation

As neural crest-derived cells, SCAPs demonstrate neurogenic differentiation capacity* in vitro* after induction. Previous reports have provided evidence that, upon stimulation with a neurogenic medium containing B27 supplement, bFGF, and epidermal growth factor (EGF), SCAPs express a variety of markers of neural precursors, neuron, and glial cells, such as nestin, neurogenin 2, musashi 1, neuronal nuclei, neuron-specific enolase, *β*III tubulin, microtubule associated protein 2, neurofilament, glial fibrillary acidic protein, 2′, 3′-cyclic nucleotide-3′ phosphodiesterase, glutamic acid decarboxylase, and neural cell adhesion molecule [2, 16, 20, 56–60]. Moreover, several studies investigate that fibrinogen 50-thrombin 50 and SFRP2 could promote neurogenic differentiation of SCAPs [61, 62].

### 4.3. Other Lineage Differentiations

The plasticity of SCAPs enables them to differentiate into other cell lineages. For example, after induction with adipogenic medium, SCAPs can form characteristic oil red O-positive lipid-containing adipocytes [1–4, 20, 60]. This phenotypic conversion is also correlated with the expression of adipocyte-specific markers, such as adipocyte fatty acid binding protein 2, peroxisome proliferator-activated receptor-*γ*2 and lipoprotein lipase [3, 4]. The ability of SCAPs to differentiate into chondrocytes* in vitro* has also been noted. Under appropriate culture conditions, SCAPs can express chondrogenic differentiation markers such as SRY-box 9 and collagen type II and form cartilage as identified by alcian blue staining [3, 4, 20, 60]. In addition, SCAPs can be induced* in vitro* to differentiate into hepatocytes, characterized by the production of urea and the expression of hepatic-specific markers, such as hepatocyte nuclear factor 1-*α*, *α*-1 fetoprotein, alanine amino transferase, and aspartate amino transferase [3, 63].

These results provide insight into the differentiation of SCAPs. However, the mechanisms underlying the directed differentiation remain unclear, which need to be further investigated.

## 5. Therapeutic Potential of SCAPs

Stem cell-based therapy is an emerging field as a promising medical treatment of multiple diseases [64]. SCAPs have the ability to differentiate into various cell types and possess low immunogenicity, which could contribute to the regeneration and repair of tissues. Hence they can be considered as an attractive alternative cell source for stem cell-based therapy.

### 5.1. Pulp-Dentin Regeneration

Irreversible pulpitis and periapical periodontitis, usually caused by dental trauma and caries, are common diseases in oral cavity. In recent years, regenerative endodontics has been a promising treatment for these diseases instead of apexification. SCAPs are characterized by a high proliferation rate and odontogenic differentiation potential, which makes them suitable for stem cell-based regeneration and producing dentin-pulp complex. After transplantation of SCAPs combined with hydroxyapatite/tricalcium phosphate (HA/TCP) scaffolds into immunocompromised mice, a layer of dentin tissue is generated on the surface of the HA/TCP [1]. When SCAPs are seeded onto synthetic scaffolds consisting of poly-D, L-lactide/glycolide, inserted into tooth fragments and transplanted into immunocompromised mice, a continuous layer of dentin-like tissue is deposited on the dentin surface and vascularized pulp-like tissue is formed in the root canal [65]. Many researchers have invented novel scaffolds for regenerative endodontics, including decellularized dental pulp [66, 67] and injectable nanofibrous microspheres [68]. Functionalized scaffolds can be used as a controlled-release device for morphogenic factors to provide a conductive microenvironment for odontogenic differentiation of stem cells and pulp-dentin regeneration [51]. In addition, scaffold-free stem cell sheet-derived pellet (CSDP) can be used in pulp-dentin regeneration. The evidence indicates that SCAPs-based CSDPs transplanted into immunocompromised mice also yield pulp-dentin-like tissue [69]. Although previous studies have demonstrated the potential of SCAPs in pulp-dentin regeneration, more researches are needed in order to achieve clinical application.

### 5.2. Bioroot Engineering

Tooth loss caused by a variety of diseases such as trauma, caries, periodontal disease, and genetic disorders can lower the quality of life. Currently, dental implants are regarded as the best clinical method for replacing missing tooth instead of fixed bridge and removable denture. However, with the development of tissue engineering and regenerative medicine, tooth regeneration has become an ideal and promising method. Some case reports show continued root development after conservative treatment of immature permanent teeth with pulp necrosis and periapical lesions. This clinical phenomenon suggests that SCAPs may survive during the process of pulp necrosis and play an important role in tooth root formation by differentiating into odontoblasts [2, 70, 71]. Sonoyama et al. have demonstrated that by using SCAPs along with the PDLSCs to regenerate a bioroot with periodontal ligament tissues. A minipig model is used, and the autologous SCAPs and PDLSCs are then seeded into a root-shaped scaffold with a postchannel in the middle, and implanted into a socket of alveolar bone. Three months later, the bioroot is formed and can support a porcelain crown to provide normal tooth function. Compared with dental implants, the bioroot is encircled with periodontal ligament tissue and has favorable biomechanical properties [1]. However, there has only been limited study of tooth root regeneration, so more researches are required to reach the potential of SCAPs in bioroot engineering.

### 5.3. Periodontal Tissue Regeneration

Periodontitis, one of the most widespread chronic infectious diseases, results in the destruction of tooth-supporting tissues and associates with many systemic diseases. Conventional treatments for periodontitis, including scaling, root planning, and periodontal flap surgery, can only alleviate the inflammation of periodontal tissues and form a long junctional epithelium instead of periodontal attachment, so alternative regeneration methods are necessary to regenerate periodontal tissues. Recently, stem cell-based therapy is considered highly promising for periodontal tissue regeneration. 12 weeks after injecting SCAPs into periodontitis animal model, clinical assessments, CT scans, and histopathology results show that SCAPs could significantly improve periodontal regeneration [72]. This study supports the concept of using SCAPs as a suitable alternative stem cell source for periodontal tissue regeneration in the future.

### 5.4. Bone Regeneration

Recently, with the development of biocompatible materials and the discovery of stem cell sources, bone tissue engineering has become an alternative approach for repairing large bone defects instead of bone grafting. As mentioned earlier,* ex vivo* expanded SCAPs have the capacity to differentiate into osteoblasts after culture in osteogenic medium. To further investigate the potential to form bone tissue, SCAPs combined with scaffolds are implanted subcutaneously into immunocompromised mice. After a period of time, ectopic bone-like tissue is generated, which contains osteocyte-like cells and osteoblast-like cells [1, 5, 19]. These results indicate the feasibility of SCAPs transplantation in the treatment of bone defects, but extensive work lies ahead in order to achieve clinical application.

### 5.5. Neural Regeneration and Repair

SCAPs derived from the cranial neural crest have the capacity to differentiate into neural cells under inductive conditions. Therefore they may be a potential cell source for the treatment of nerve injuries. To regenerate nerve tissue, researchers have attempted to cultivate SCAPs in 3D organotypic culture, which eventually generate 3D cell-based nerve-like tissue with axons and myelin structures* in vitro* [56]. Moreover, in a rat hemisection model of spinal cord injury, transplantation of apical papilla tissue into the lesion site can improve gait and reduce glial reactivity [73]. Another study indicates that transplanted SCAPs can protect spinal cord neurons and promote functional recovery after spinal cord injury [74]. Additionally, in a rat sciatic nerve injury model, SCAPs also exert neuroprotective effects on the dorsal root ganglia neurons and stimulate axon regeneration [75]. Previous reports suggest that SCAPs are able to secrete neurotrophic factors such as nerve growth factor, brain derived neurotropic factor, neurotrophin-3, and activin-A [76–78]. Taken together, these observations seem to indicate that SCAPs are excellent candidates for stem cell-based therapy in central and peripheral nerve injuries.

### 5.6. Angiogenesis

Ischemic disease is a major cause of disability and death. Currently, stem cell-based therapeutic angiogenesis is an alternative treatment for ischemic diseases. In recent years, the transdifferentiation capacity of SCAPs into endothelial cells has been evaluated. After exposure to angiogenic medium, SCAPs can undergo morphological changes to endothelial cells, express higher levels of several angiogenesis-related genes, and form capillary-like structures* in vitro* [79]. Furthermore, a series of experiments have shown that SCAPs possess the ability to promote angiogenesis. SCAPs can secrete several proangiogenic molecules that are able to improve the angiogenic potential of endothelial cells, such as angiogenin, VEGF, and insulin-like growth factor binding protein 3 [79, 80]. A chorioallantoic membrane assay demonstrates that SCAPs also stimulate new blood vessel formation in an* in vivo* setting [80]. Especially under hypoxic conditions, the proangiogenic effect of SCAPs is increased [81, 82]. These results indicate that, due to their angiogenic potential, SCAPs are attractive options for stem cell-based therapeutic angiogenesis.

### 5.7. Immunotherapy

In addition to multilineage differentiation capacity, SCAPs possess immunomodulatory functions, which indicate that they may be a potential immunotherapeutic tool for treating autoimmune and inflammation-related diseases. Previous research confirms that SCAPs express low levels of immunological molecules, such as swine leukocyte antigen (SLA) class I molecules and SLA class II DR molecules in a minipig model. Moreover, SCAPs are capable of inhibiting T cell proliferation* in vitro* through an apoptosis-independent mechanism [83]. From these studies, it is apparent that SCAPs have immunosuppressive properties, but the exact mechanisms remain unknown. So there are still challenges to be solved before SCAPs can be applied clinically.

## 6. Conclusions

In conclusion, the isolation of SCAPs from dental tissue along with discovery of their properties has provided a conceptual framework of their nature and potential application. However, several aspects of SCAPs biology remain in question and unsettled, which include the identity, nature, standardization of isolation and culture protocols, cell banking procedures, and* in vivo* use for therapy. More progress on stem cells made in nondental tissues will help in adopting research strategies used in SCAPs. Simultaneously, a better understanding of the novel population of postnatal somatic stem cells could facilitate the full utilization of stem cells in clinical practice.

## Figures and Tables

**Table 1:** Marker expression in SCAPs.

Positive markers	Negative markers
CD13, CD24, CD29, CD44, CD49, CD51,	CD14, CD18,
CD56, CD61, CD73, CD90, CD105,	CD34, CD45,
CD106, CD146, CD166, STRO-1, Oct3/4,	CD117, CD150
Sox-2, Nanog, Notch 3, vimentin, survivin	

Abbreviations: CD, Cluster of differentiation; Oct3/4, octamer binding transcription factor-3/4; Sox-2, sex determining region Y-box 2; Nanog, nanog homeobox.
